# *Enterococcus faecium* PNC01 isolated from the intestinal mucosa of chicken as an alternative for antibiotics to reduce feed conversion rate in broiler chickens

**DOI:** 10.1186/s12934-021-01609-z

**Published:** 2021-06-28

**Authors:** Yang He, Xuan Liu, Yuanyang Dong, Jiaqi Lei, Koichi Ito, Bingkun Zhang

**Affiliations:** 1 State Key Laboratory of Animal Nutrition, Department of Animal Nutrition & Feed Science, College of Animal Science & Technology, China Agricultural University, Haidian District, Beijing, 100193 China; 2 College of Veterinary Medicine, China Agricultural University, Haidian District, Beijing, 100193 China; 3 Department of Food and Physiological Models, Graduate School of Agricultural and Life Sciences, The University of Tokyo, 3145 Ago, Kasama, Ibaraki 319-0206 Japan

**Keywords:** *E. faecium* PNC01, Feed conversion rate, Antibiotics, Intestinal mucosa, Broiler chickens

## Abstract

**Background:**

The development and utilization of probiotics had many environmental benefits for replacing antibiotics in animal production. Bacteria in the intestinal mucosa have better adhesion to the host intestinal epithelial cells compared to bacteria in the intestinal contents. In this study, lactic acid bacteria were isolated from the intestinal mucosa of broiler chickens and investigated as the substitution to antibiotic in broiler production.

**Results:**

In addition to acid resistance, high temperature resistance, antimicrobial sensitivity tests, and intestinal epithelial cell adhesion, *Enterococcus faecium* PNC01 (*E. faecium* PNC01) was showed to be non-cytotoxic to epithelial cells. Draft genome sequence of *E. faecium* PNC01 predicted that it synthesized bacteriocin to perform probiotic functions and bacteriocin activity assay showed it inhibited *Salmonella* typhimurium from invading intestinal epithelial cells. Diet supplemented with *E. faecium* PNC01 increased the ileal villus height and crypt depth in broiler chickens, reduced the relative length of the cecum at day 21, and reduced the relative length of jejunum and ileum at day 42. Diet supplemented with *E. faecium* PNC01 increased the relative abundance of Firmicutes and *Lactobacillus*, decreased the relative abundance of Bacteroides in the cecal microbiota.

**Conclusion:**

*E. faecium* PNC01 replaced antibiotics to reduce the feed conversion rate. Furthermore, *E. faecium* PNC01 improved intestinal morphology and altered the composition of microbiota in the cecum to reduce feed conversion rate. Thus, it can be used as an alternative for antibiotics in broiler production to avoid the adverse impact of antibiotics by altering the gut microbiota.

**Graphic Abstract:**

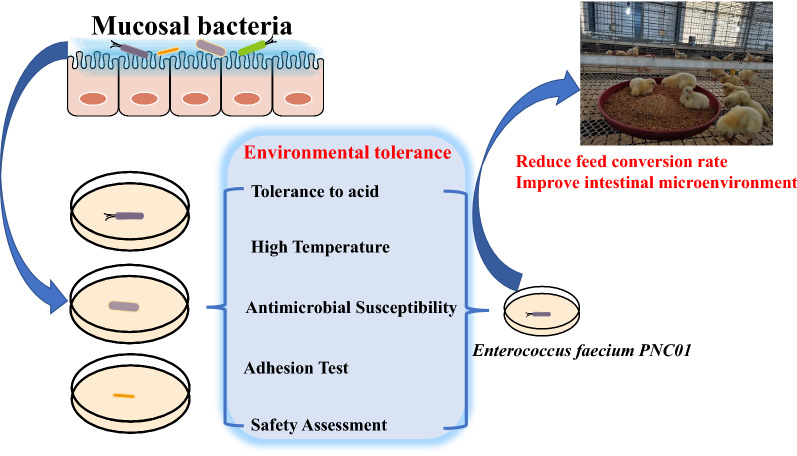

**Supplementary Information:**

The online version contains supplementary material available at 10.1186/s12934-021-01609-z.

## Background

The excessive use of antibiotics in animal husbandry had led to the widespread of antibiotic resistant bacteria and antibiotic resistant genes [1], which had seriously threatened the effectiveness of antibiotics and public health. Statistics showed the antibiotic consumption in animal husbandry was 148 mg/kg poultry, 45 mg/kg cattle, and 172 mg/kg pigs [2]. China consumed more than 91,000 tons of antibiotics for livestock in 2015 [3]. The residual antibiotics contaminated the environment with feces [4]. The most effective approach to avoid the adverse effects of antibiotics was to reduce the initial use of antibiotics. Therefore, the European Union, China and India have made policies to ban the use of antibiotics in feed [2, 5]. China as the largest consumer of veterinary antibiotics has banned the addition of antibiotics in animal feed from 2020. The ban on antibiotics reduced the animal growth performance, leading to environmental pollution caused by nutrients emission with feces and urine [6]. Therefore, the development of green and sustainable antibiotic alternatives was particularly important to improve the production performance of animal and their intestinal health.

Broiler feed conversion rate (FCR) is the rate of feed intake to body weight gain [7]. Reducing the FCR was of great significance for improving animal production performance and reducing environmental pollution [8, 9]. The main purpose of adding antibiotics to feed was to promote intestinal health, improve nutrients absorption in the gut and prevent pathogenic bacteria infection [10]. Colistin sulfate reduced the intestinal wall thickness to improve nutrient absorption and reduced FCR [11]. Probiotics would not introduce other hazardous substances into the environment like antibiotic substitutes, so they had important application prospects. The regulation of the intestinal bacterial community through nutrition reduced the FCR [12]. Study has showed that *Lactobacillus* spp. improved the crude protein retention and meat quality in animals [13, 14]. Short-chain fatty acids such as butyrate produced by intestinal microorganisms fermentation in chickens improved the proliferation of intestinal epithelial cells [15]. The development and utilization of the existing beneficial microorganisms from the gut had great potential to replace antibiotics.

*Enterococcus faecium* was the first to be used as a probiotic feed additive and permitted by the European Union and FDA [16]. *Enterococcus faecium* has been shown to improve the intestinal immunity and the jejunal mucus secretion in the broiler chickens [7, 17]. *Enterococcus faecium* improved the resistance to pathogenic bacteria in animals [18, 19]. The poultry gut was rich in lactic acid bacteria (LAB), which has be used as an important biological resource for the isolation of probiotics [20]. A large number of LAB has been isolated from the intestinal contents of animals and been used as probiotics through the Man Rogosa Sharpe (MRS) agar medium [21, 22]. As the adhesion of probiotics to the intestinal epithelium is prerequisite for their function and adhesion colonization has host specificity [19]. The bacteria in the intestinal mucosa should be paid more attention because they have better adhesion ability than bacteria in chyme. The feeding effect of homologous probiotics is better than heterologous probiotics [23]. Therefore, it is necessary to systematically evaluate the effects of isolated potential probiotics from the homologous intestinal mucosa on growth performance and intestinal microbiota in broiler chickens.

Therefore, the purpose of this study was to isolate and identify LAB from the intestinal mucosa of broiler chickens and to select probiotics with the potential to alternative antibiotics through in vitro evaluation. The biological function of the bacteria was predicted through draft genome sequencing. The effects of the selected bacteria on the growth performance, intestinal morphology, immune organs and intestinal bacteria in broiler chickens, providing new applications for the use of microbial resources instead of antibiotics.

## Results

### Isolation of LAB

All LAB species obtained from gut of broiler chicken were identified by V1–V9 region sequencing of 16S rDNA. A total of 70 LAB from the species of *Pediococcus pentosaceus*, *Lactobacillus salivarius*, *L. reuteri*, and *Enterococcus faecium* were obtained. Among them, 13 strains were isolated from *Pediococcus pentosaceus*, 55 strains were isolated from *Lactobacillus salivarius*, 1 strain was isolated from *Lactobacillus reuteri* and 1 strain was isolated from *Enterococcus faecium*. The characteristic morphologies of colony and cell of isolated LAB are showed in Additional file 1: Table S1.

### Tolerance to Acid, High Temperature and Antimicrobial Susceptibility Assay

**Fig. 1:**
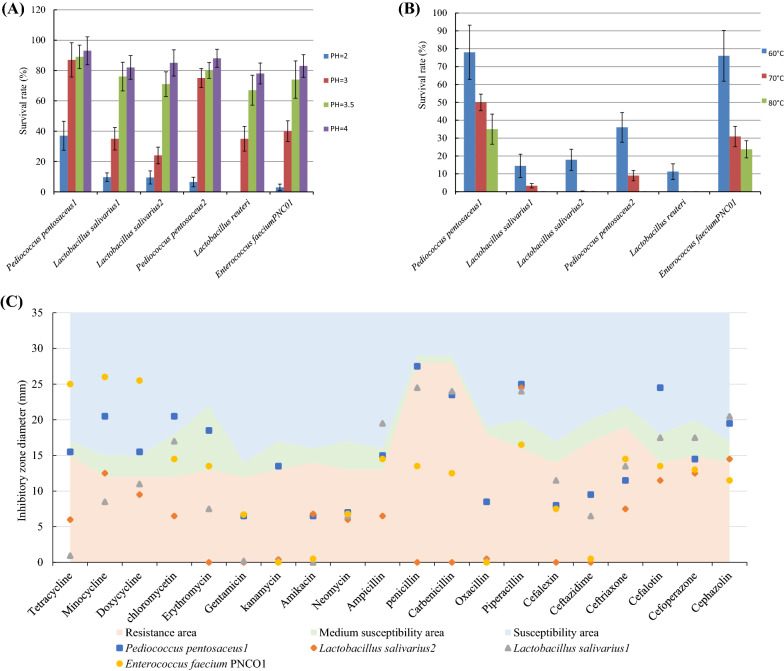
The survival rate of isolated lactic acid bacteria to pH (**A**) and temperature (**B**). The inhibitory zone diameter of isolated lactic acid bacteria to antibiotics susceptibility (**C**), and the susceptibility area were performed according to Clinical and Laboratory Standards Institute

On the bases of proliferation efficacy (Additional file 1: Table S1), six strains (2 strains of *Pediococcus pentosaceus* (*P. pentosaceus*) named *P. pentosaceus* 1 and *P. pentosaceus* 2, 2 strains of *Lactobacillus salivarius* (*L. salivarius*) named *L. salivarius* 1 and *L. salivarius* 2, 1 strain of *Lactobacillus reuteri* (*L. reuteri*) and 1 strain of *Enterococcus faecium* named *E. faecium* PNC01) were selected for further analysis. In the tolerance to acid, the *L. reuteri* lost viability at pH 2 for 4.5 h (Fig. 1A). In the tolerance to high temperature, after treatment at temperature of 70 ℃, *L. salivarius*2 and *L. reuteri* lost viability, and *L. salivarius1* and *P. pentosaceu2* only survived 3.3% and 9%, respectively (Fig. 1B). Based on the result of tolerance to acid and heat, *P. pentosaceus1, L. salivarius1*, *L. salivarius2* and *E. faecium* PNC01 were selected for further analysis, considering the loss of viability of *L. reuteri* and *L. salivarius*2 after exposure to pH 2 and temperature 70 ℃.

*P. pentosaceus* 1 was the most sensitive to antibiotics, showed resistance toward 10 antibiotics, and was sensitive to 6 antibiotics (Fig. 1C). The *L. salivarius* 2 exhibited strong antibiotic resistance, was able to resist 17 antibiotics and was sensitive to only 1 antibiotic (Fig. 1C). Based on the result of antimicrobial susceptibility assay, *P. pentosaceus*1 and *E. faecium* PNC01 were selected for further analysis, considering they were sensitive to antibiotics.

### Adhesion test and safety assessment

**Fig. 2:**
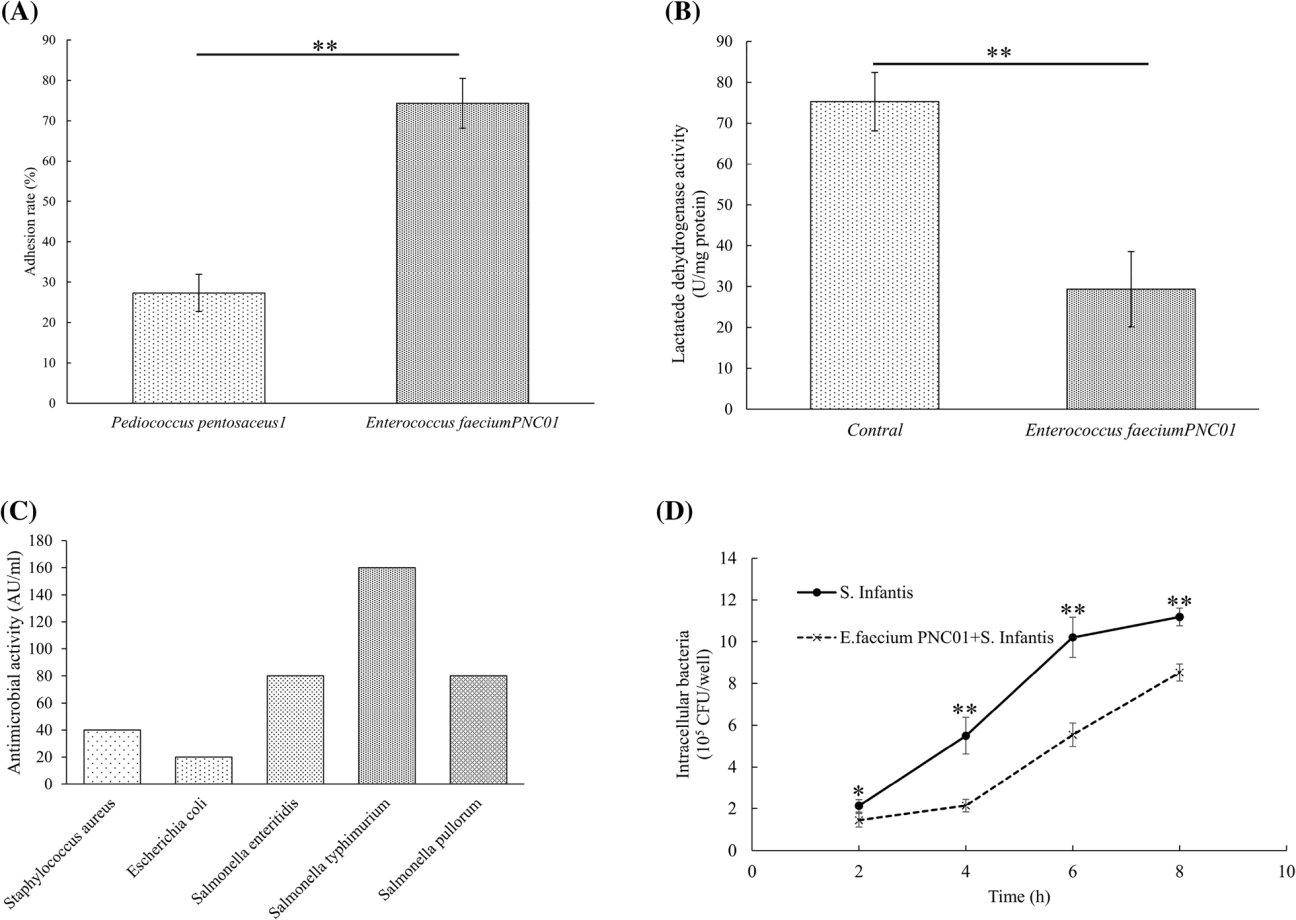
The Adhesion rate of *P. pentosaceus1* and *E. faecium* PNC01 to Caco-2 cell line (**A**). The effects of *E. faecium* PN01 on the lactatede dehydrogenase activity (**B**). Antibacterial activity of bacteriocins against pathogenic bacteria (**C**). *Enterococcus faecium* PN01 inhibited *Salmonella* typhimurium from invading intestinal epithelial cells. ** indicated very significant difference (*P* < 0.01)

The adhesion rates of the selected bacteria to intestinal epithelial cell are showed in Fig. 2. The adhesion efficiency of *E. faecium* PNC01 was significantly higher than that of *P. pentosaceus1*, and their adhesion efficiency was 74.3% and 27.3%, respectively (Fig. 2A). Further, the cytotoxicity of selected strain was tested, the lactate dehydrogenase activity in Caco-2/*E. faecium* PNC01 co-culture supernatant was lower than Caco-2 individually cultured control group. The *E. faecium* PNC01 was safe to be tested in an animal study (Fig. 2B). The *E. faecium* PNC01 had different antibacterial activities against the detected pathogenic bacteria, especially it had the highest antibacterial activity to *Salmonella* typhimurium and the least antibacterial activity to *Escherichia* coli (Fig. 2C). At the same time, *E. faecium* PNC01 significantly inhibited the invasion of Salmonella into intestinal epithelial cells (Fig. 2D).

### Draft genome sequence of E. faecium PNC01

A total of 7,647,355 paired-end raw reads with 1.14G bases were generated. Then 7,450,427 paired reads with 1.12G bases of clean data were retained through removing the adapter sequences and filtering the low-quality data. The genome length of *E. faecium* PNC01 was 2,186,577 bases with a G + C content of 38.1%, contained 2463 genes about 85.4% of genome. A total of 41 tRNA genes and 3 rRNA operons gene were predicted from the contigs. The phylogenetic tree analysis showed that *E. faecium* PNC01 was significantly different from other known *Enterococcus faecium* (Additional file 1: Figure S1), and the secondary metabolite prediction analysis showed that the *E. faecium* PNC01 could synthesize bacteriocin. The subsystem description of *E. faecium* PNC01 showed it had metabolic function (Additional file 1: Figure S2). The isolated bacterium encoded no pathogenicity island or other virulence determinants by predicting the genome draft.

### Growth performance and feed intake of broiler chickens

**Fig. 3:**
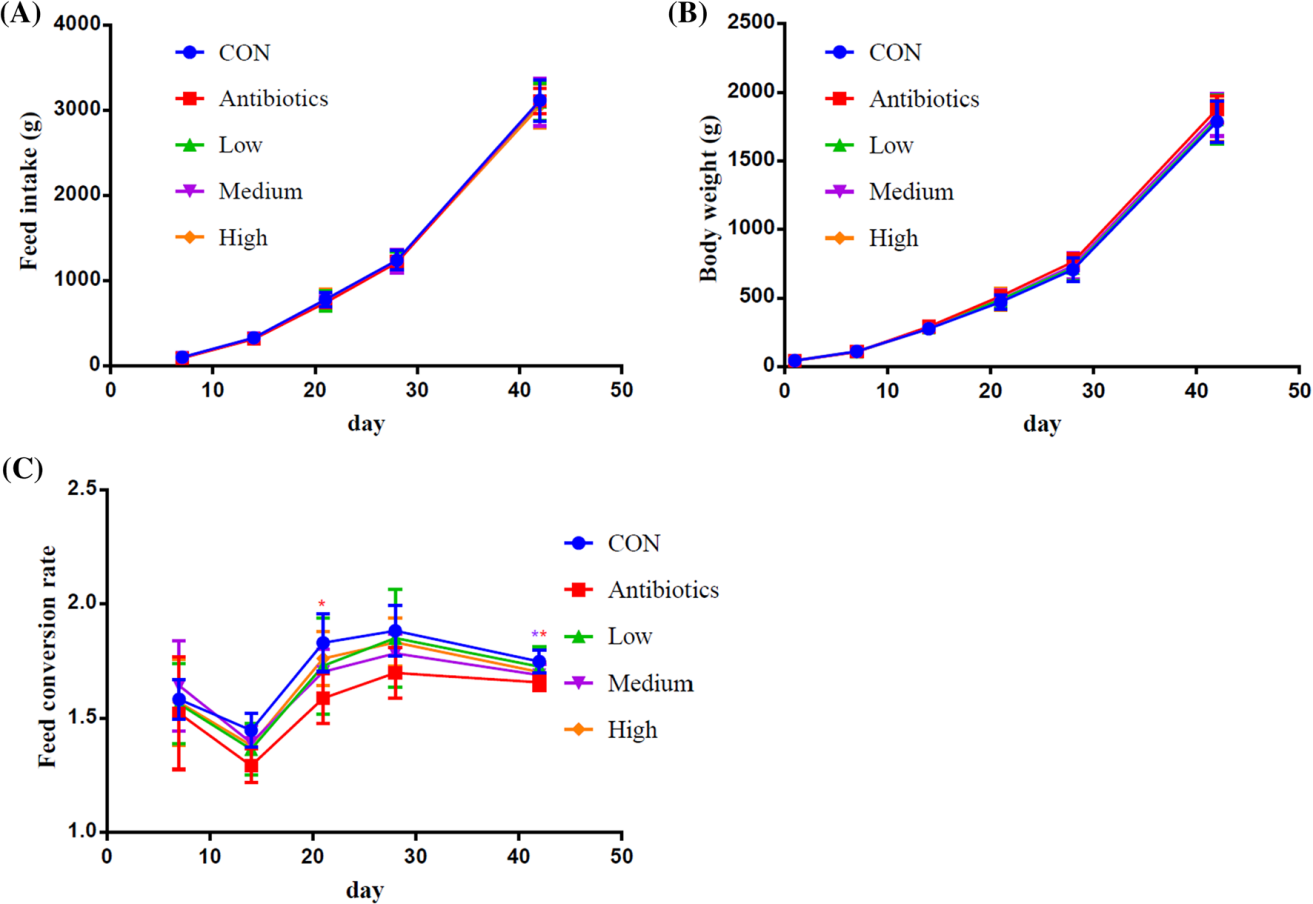
Effect of Antibiotics and *E. faecium* PNC01 on the production performance of broiler chickens. The effect of dietary treatment on the feed intake (**A**) and body weight (**B**) of broilers was not significant, but the antibiotics group (red *) and the medium-dose *E. faecium* PNC01 group (purple *) can significantly reduce the feed conversion rate (**C**), compared with the control group

The effects of antibiotics and *E. faecium* PNC01 on the growth performance and feed intake in broiler chickens are presented in Fig. 3. The antibiotics and *E. faecium* PNC01 had no effect on the feed intake and growth performance regardless of the growth stage. At day1-21, compared with the control group (CON), dietary supplement with antibiotics or *E. faecium* PNC01 at low (1 × 10^8^ CFU/kg feed) and medium (1 × 10^9^ CFU/kg feed) levels significantly reduced FCR in broiler chickens. At day 1–42, antibiotics and medium groups reduced FCR, while high (1 × 10^10^ CFU/kg feed) or low groups did not significantly reduce FCR.

**Fig. 4:**
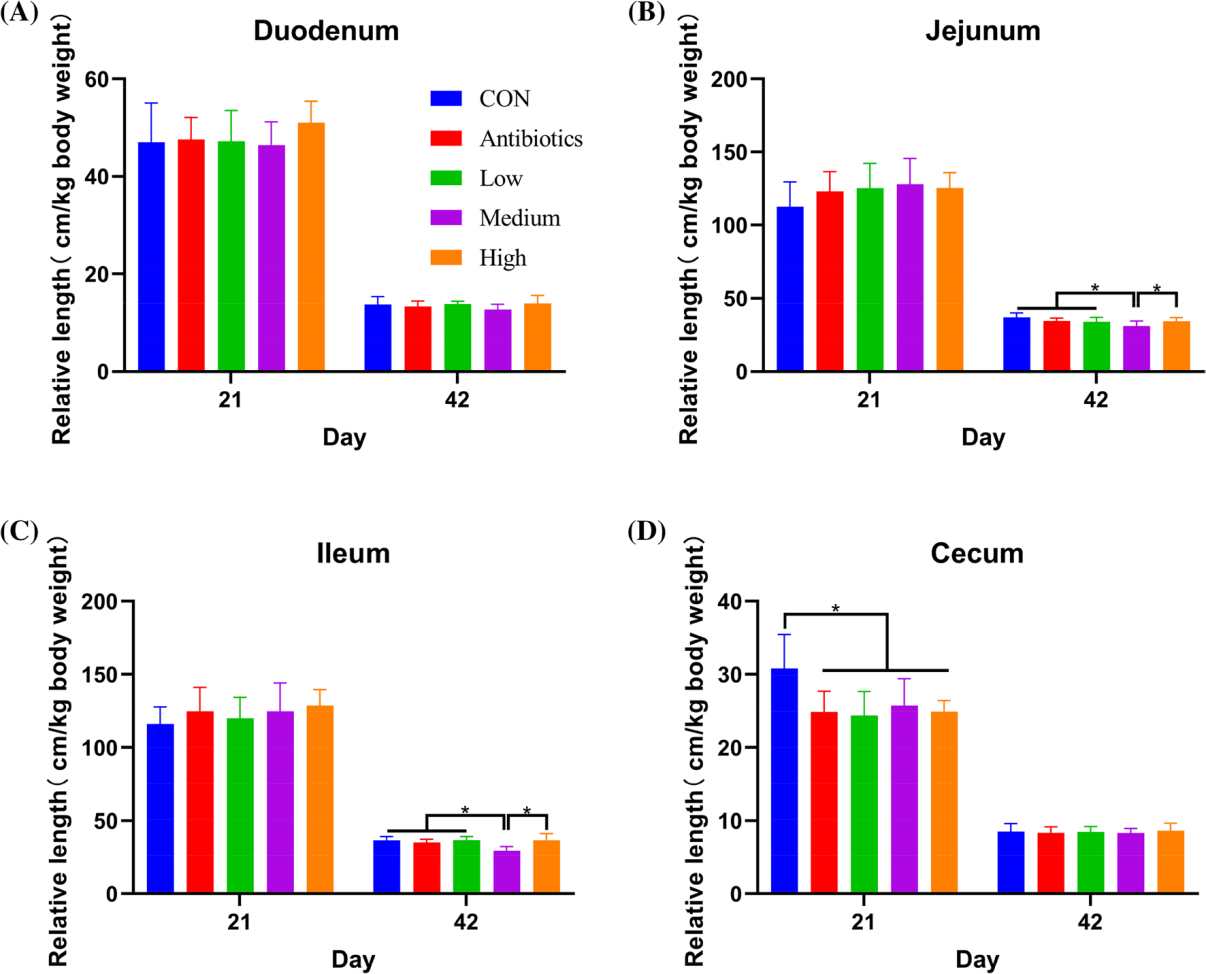
Effect of antibiotics and *E. faecium* PNC01 on the relative length of duodenum (**A**), jejunum (**B**), ileum (**C**) and cecum (**D**) on day 21 and 42 of broiler chickens. * indicated significant difference (*P* < 0.05)

The relative length of the jejunum and ileum in control group on day 42 was significantly shorter than that in other groups (Fig. 4). Adding antibiotics and *E. faecium* PNC01 significantly reduced the relative length of the cecum on day 21 (Fig. 4D). There was no significant difference in the immune organs (thymus, spleen and bursa of fabricius) indexes among the treatment groups (Additional file 1: Table S2).

### Intestinal morphology

**Fig. 5:**
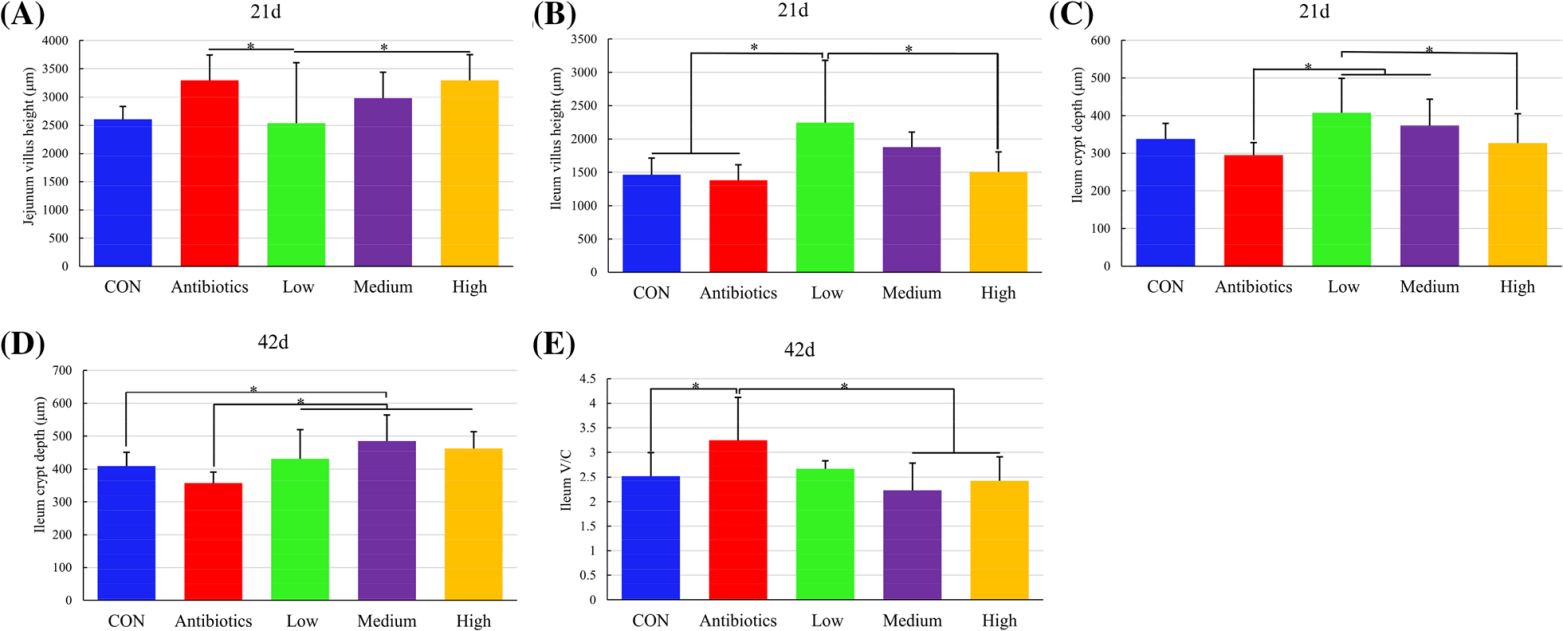
Effect of antibiotics and *E. faecium* PNC01 on jejunal and ileal morphology of broiler chickens. * indicated significant difference (*P* < 0.05). V/C: The ratio of Villus height to Crypt depth

The effects of antibiotics and *E. faecium* PNC01 on intestinal morphology are showed in Fig. 5. At day 21, the jejunal villus height in the antibiotics and the high groups was significantly higher than that in the low group (Fig. 5A). At day 21, the ileal villus height in the low group was higher than that in the other groups except for the medium group (Fig. 5B). The crypt depth in the low group was higher than that in the antibiotics and the high groups, and the antibiotics group was lower than the medium group (Fig. 5C). At day 42, the crypt depth in the antibiotics group was lower than that in the medium and high groups, and the medium group was significantly higher than the CON group (Fig. 5D). At day 42, the V/C in the antibiotics group was significantly higher than that in the CON, medium and high groups (Fig. 5E). Other intestinal morphologies with insignificant differences are shown in Additional file 1: Figure S3.

**Fig. 6:**
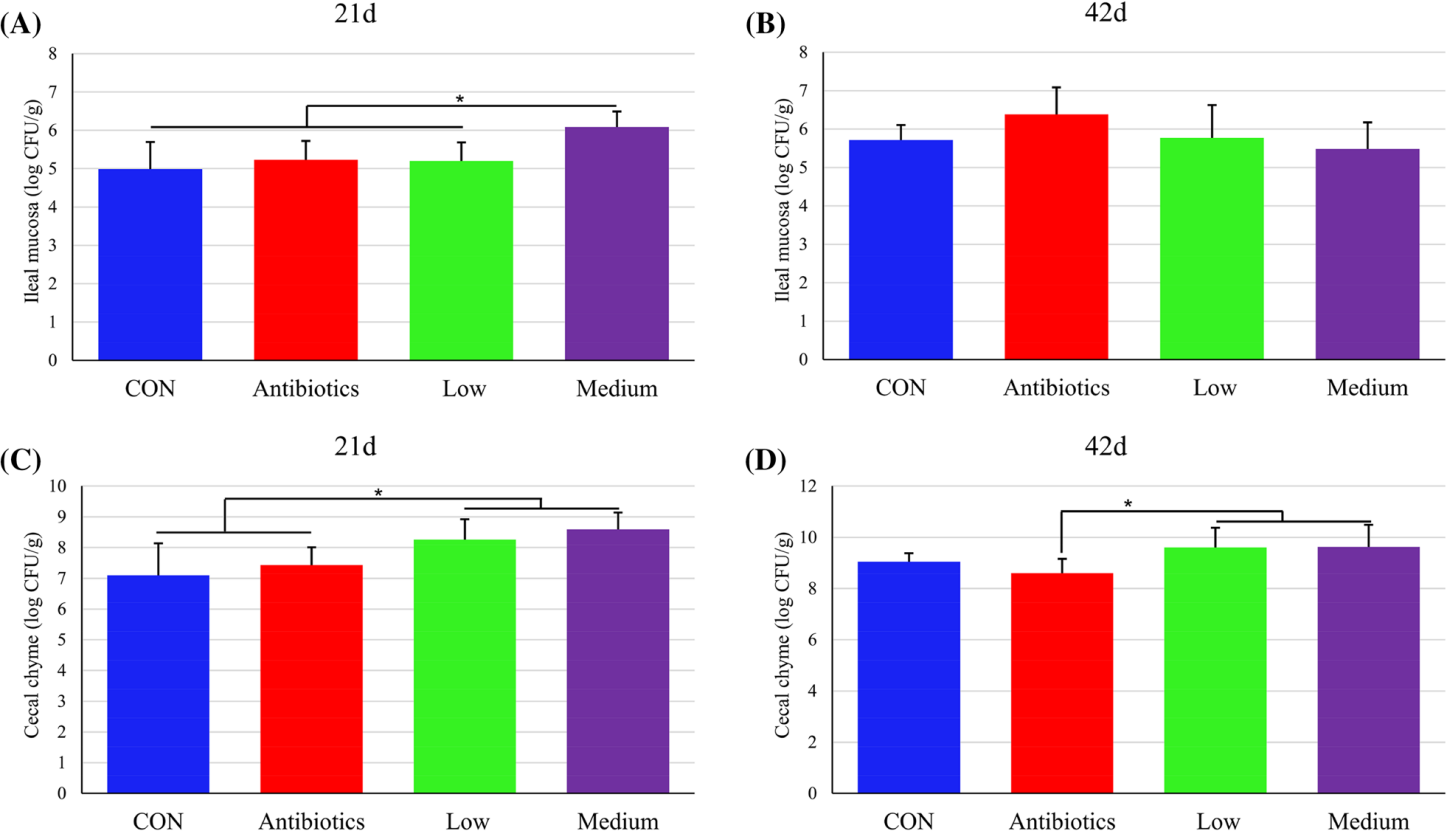
Effect of antibiotics and *E. faecium* PNC01 on the intestinal lactic acid bacteria of broiler chickens. The number of lactic acid bacteria in the ileal mucosa at day 21 (**A**) and 42 (**B**). The number of lactic acid bacteria in colon chyme at day 21 (**C**) and 42 (**D**). * indicated significant difference (*P* < 0.05)

The effect of dietary treatment on the content of LAB in the intestinal chyme and mucosa of chicken is presented in Fig. 6. At day 21, the ileal mucosa LAB number in the medium group was significantly higher than in the other groups (Fig. 6A). However, the difference was not significant at 42 day (Fig. 6B). The cecal chyme LAB content in the *E. faecium* PNC01 groups was higher than that in the CON and the antibiotics groups (Fig. 6C). At day 42, the cecal chyme LAB number in the antibiotics group was significantly lower than the *E. faecium* PNC01 added groups (Fig. 6D). The number of other intestinal LAB with insignificant differences is shown in Additional file 1: Figure S4.

### 16sDNA sequencing of cecal bacteria

**Fig. 7:**
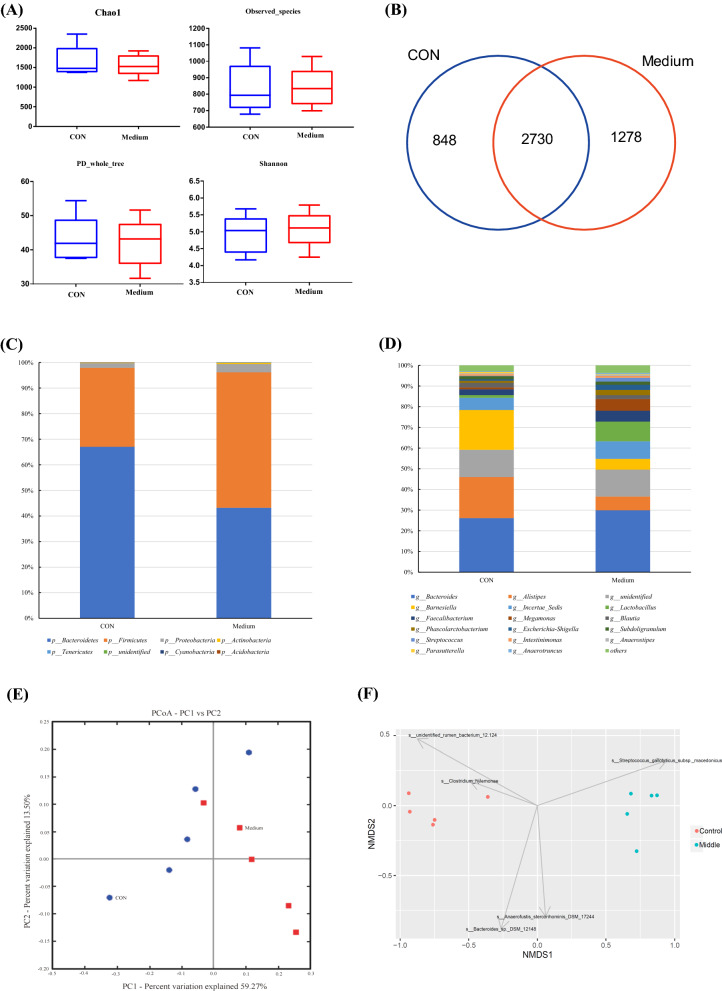
The effects of intestinal bacterial community on the cecal bacterial community diversity indexes of chao1, observed_species, PD_whole_tree, and Shannon (**A**). Venn diagramof the OTUs (**B**). The effects of intestinal bacterial communities on the relative abundances of ceccal bacteria at the phylum level (**C**) and genus level (**D**). Principal component analysis (PCA) of cecal bacteria communities based on weighted UniFrac distances (**E**). Non-metric multi-dimensional scaling (NMDS) analysis of cecal bacteria (**F**)

The cecal bacterial community in the medium and the CON groups were sequenced and compared as the medium group achieved better antibiotic replacement effects. A total of 364,566 sequencing reads from 17,608 to 52,647 tags per sample were obtained after demultiplexing and quality filtering. The sequences were clustered into 856 OTUs at 97% sequencing identity. The alpha diversity indexes in the two groups had no significant difference (Fig. 7A). The Venn chart showed that there were 2730 OTUs of microorganisms shared between the two groups. The unique OTUs in the control group and the medium group were 848 and 1278 respectively (Fig. 7B). This showed that the two groups had huge differences in the composition of the bacteria.

At phylum level, Bacteroidetes, Firmicutes, and Proteobacteria were the predominant bacteria, accounting for 55.1%, 41.9%, and 2.5%, respectively (Fig. 7C). The relative abundance of Bacteroidetes in the CON group was significantly higher than that in the CON group, while the relative abundance of Firmicutes in the medium group was significantly higher than that in the CON group. but the relative abundance of Proteobacteria had no difference between the two groups.

At genus level, *Bacteroides* spp., *Alistipes* spp., and *Barnesiella* spp. were the major predominant genera accounting for 28.2%, 13.3%, and 12.3%, respectively (Fig. 7D). *Alistipes* spp. was higher in the CON group as compared to medium group, whereas *Barnesiella* spp. was significantly lower in CON group as compared with the medium group. *Lactobacillus* was dominant genus in medium group, which was significantly higher in medium group as compared to the CON group. The principal coordinate analysis (PCoA) of the weighted unifrac distance metric and non-metric multi-dimensional scaling (NMDS) analysis indicated that cecal bacterial community between the two groups was difference as presented in Fig. 7E and F.

## Discussion

Understanding the effects of feeding practices on animal health, growth performance, and gut microbiota using alternatives (such as probiotics) is important for strategies to reduce antibiotic use in poultry production and for reducing environmental pollution. Environmental microorganisms had huge diversity, and the development and utilization of these microorganisms had great potential for sustainable agricultural development and green-friendly production [24].

The temperature of feed up to 70 ℃ during processing, and the pH value of glandular stomach in poultry is about 2. The tolerances to extreme environmental conditions like high temperature and low pH were indispensable for probiotics to perform probiotic functions [25]. Probiotics with high heat resistance were conducive to industrial processing, storage and transportation [26]. Survival of probiotics in the acidic environment of the gastrointestinal tract was a precondition for probiotics survive passing through the stomach [27]. In this study, the genus of *P. pentosaceus* and *E. faecium* had the potential to be used as probiotics in previous reports [28].

Large-scale broiler farming has aggravated the spread of antibiotic resistant bacteria, leading to a gradual decrease in the antibiotic sensitivity of pathogenic bacteria [29]. Previous study has reported *Bacillus* probiotics posed a potential risk into probiotic species from the genus *Bacillus* as it contained antimicrobial resistance genes [30]. Therefore, the selection of probiotics should consider their antibiotics sensitivity to avoid introduce resistance genes into the host gut. The selected probiotics were found to resistant multiple antibiotics in present study. The diameters of inhibitory zone of LAB for aminoglycosides and penicillins were larger than that of tetracyclines, chloramphenicols, macrolides, β-lactams and cephalosporins. Those results were consistent with previous study that LAB were resistant to aminoglycosides and penicillins [31]. *P. pentosaceus1* and *E. faecium* PNC01 were selected for the further step analysis as they had a higher sensitivity to antibiotics.

The adhesion of probiotics to intestinal epithelial cells can exert probiotic functions in a variety of ways. Such adhesion competitively inhibited the adhesion of pathogenic bacteria to the host intestine epithelia, and the barrier formed by the adhesion of probiotics reduced the invasion of pathogenic microorganisms [32]. The adhered LAB to the intestinal epithelial cells of chickens and pigs reduced the invasion of *Campylobacter* [19]. *E. faecium* synthesized a bacteriostatic agent which prevented the tight junction disruption caused by *Listeria monocytogenes* and improved the immune function of intestinal epithelial cells [33]. Probiotics' adhesion to intestine epithelia was a precondition for their growth and function in the gut. In this study, the adhesion of *E. faecium* PNC01 was 2.7 times of *P. pentosaceus1*. Intestinal epithelial cell injury increased the lactate dehydrogenase activity in the supernatant [34]. *E. faecium* PNC01 reduced extracellular lactate dehydrogenase activity, indicating that it reduced cell damage and was safe for intestinal cells. Therefore, the *E. faecium* PNC01 was finally chose to instead of antibiotics in animal study.

The draft genome sequence of *E. faecium* PNC01 showed it had full metabolic function. Study has showed that the genome length of LAB was 1.8–3.35Mbp, and the percentage of C + G was usually less than 50% [35], *E. faecium* PNC01 also had the similar characteristics. The secondary metabolite prediction analysis indicated that it synthesized bacteriocin and had potential probiotic effects [36]. This was confirmed by the ability of bacteriocin activity to inhibit the growth of tested pathogenic bacteria.

In the animal study, the reduced FCR in the antibiotic supplement group and the *E. faecium* PNC01 group indicated that that adding antibiotics or *E. faecium* PNC01 in diet of chicken produced more chicken meat when consumed the same amount feed. Reducing FCR effectively increased energy conversion and protein deposition [37]. Study has showed that a lower FCR had an important role in promoting the sustainable development of animal husbandry and reducing environmental pollution [9]. General, a lower feed intake in broiler chickens can reduce FCR. However, the mechanism of antibiotics and *E. faecium* PNC01 reduced FCR in this study may through other approaches. Because they had no significant difference on feed intake and growth performance. At the same time, based on the production indexes, it can be concluded that adding *E. faecium* PNC01 at concentration 1 × 10^9^ CFU/kg feed replaced antibiotics and reduced the impact of antibiotics and their metabolites on the environment.

The intestine is the main tissue for nutrients digestion and absorption, and its development directly affects the growth performance of animals. Study has shown that intestinal epithelial area was positively related to the nutrient digestion and absorption. Probiotics reduced the intestinal length, reduced the consumption of nutrients and energy by the intestinal tissue itself, and then reduced the FCR [38]. Poor nutrient absorption compensatory increased the growth of the intestine. Therefore, the addition of antibiotics increased the absorption rate per unit intestinal length. Although a longer intestinal length was beneficial to the digestive and absorption functions of animals, the digestive and absorption functions mainly depended on the morphological structure of the intestine [39]. Therefore, the decrease in intestinal length and the increase in production performance were not contradictory. The mechanism needs further research and verification. In addition, the addition of antibiotics and probiotics had no significant effect on the immune organ (thymus, spleen and bursa of fabricius) indicators of chickens, indicating that they did not adversely affect broiler chickens.

The intestinal morphology had a decisive influence on the intestinal epithelial area, and the raised villus greatly increased the surface area of intestinal epithelium that increased nutrient absorption [40]. *E. faecium* PNC01 reduced FCR by increasing the villus height of the intestinal epithelium, which was consistent with the previous results that LAB increased the villus surface area [13]. The intestinal crypts mainly secreted digestive enzymes, and the crypts became shallower in depth after differentiation and maturation. Increasing the V/C was benefit to improve intestinal function [11]. In our study, the antibiotics reduced feed conversion mainly by increasing intestinal secretion. The previous results also proved that the antibiotics mainly increased the V/C to make the intestinal wall thinner and to improve absorption [41].

There were a variety of LAB in the intestine of broiler chickens and other animals. The probiotic effect of LAB was main to reduce the number of intestinal pathogenic bacteria and improv the intestinal morphology. It reduced the number of *E. coli* and *Campylobacter* in the gut [19]. In vivo and in vitro studies have shown that it improved the tight junctions between the intestinal epithelial cells, reduced the permeability of intestinal epithelial cells and thus inhibited the invasion of pathogenic bacteria [21, 42]. In *vitro* studies had shown that *E. faecium* PNC01 inhibited *Salmonella* from invading intestinal epithelial cells and had the activity of inhibiting a variety of pathogenic bacteria. In this study, the addition of *E. faecium* PNC01 to animal feed increased the content of LAB in the ileal mucosa and cecal chyme, indicating that this bacterial strain had a good ability to adhere and colonize.

Microorganisms were of great significance in promoting the sustainable development of agriculture [43]. Intestinal microorganisms as the largest source of microorganisms in the animal body, played an important role in improving feed utilization efficiency and reducing fecal emissions [44]. *E. faecium* PNC01 had no effect on the alpha diversity indexes of the cecal bacteria, indicating that the bacteria had no effect on the number of OTUs present of the intestinal bacteria. Previous studies have shown that adding probiotic in a non-pathological state did not significantly improve the diversity of intestinal microbiota and the steady state of the intestinal environment, which was consistent with this results [45, 46]. Bacteroidetes and Firmicutes have been reported to be the main dominant bacteria in the gut of poultry [11]. In this study, *E. faecium* PNC01 increased the relative abundance of Firmicutes, which can promote host energy metabolism. This also explained that *E. faecium* PNC01 reduced FCR in broilers. Study has shown that *Alistipes* spp. promoted energy metabolism in high fat diets [47], which was consistent with the result in this study. The increase of *Lactobacillus* spp. in lieal mucosa was also in meet with the expected results that feed supplementation with adhesive *Enterococcus faecium* increased the number of *Lactobacillus* spp. in this study. Although feeding *E. faecium* PNC01 did not cause it to become the dominant bacteria, it mainly improved the probiotic effect by increasing the relative abundance of *Lactobacillus* spp. Therefore, *E. faecium* PNC01 regulated the intestinal microenvironment to reduce the FCR of broiler chicken and achieved the effect of replacing antibiotics.

## Conclusions

Overall, *E. faecium* PNC01 isolated from broiler intestinal mucosa was selected as a substitute for antibiotics in broiler chicken feed. Diets supplementary with *E. faecium* PNC01 at 1 × 10^9^ CFU/kg feed had the same effect with antibiotics that they reduced FCR in broiler chickens. *E. faecium* PNC01 increased intestinal villus height, crypt depth, and LAB number in the ileal mucosa. In the intestinal bacteria, *E. faecium* PNC01 increased the relative abundance of the Firmicutes phylum. This study provided a practical basis for the development and use of probiotics instead of antibiotics in poultry farming to reduce the FCR by altering the intestinal microbiota.

## Methods

### Isolation of LAB

A 21-day-old male Cobb broiler chicken with the largest body weight (835 g) in the cage was selected for the isolation of bacteria at October 2014. All the broilers were raised at the Zhuozhou Poultry Experimental Farm of China Agricultural University in Dongchengfang Town, Zhuozhou City, Hebei Province (115°51′50′′ E, 39°28′37′′ N). The jejunal, ileal and cecal segments of broiler chickens were thrown longitudinally and washed three times in sterilized phosphate buffered saline (PBS) to remove the contents. The intestinal epithelial mucosa was scraped by a glass slide and placed in a sterile centrifuge with the sterilized phosphate buffered saline (PBS), then settled for 30 min after shaking. The supernatant was collected and serially diluted ten-fold using PBS from 10^–1^ to 10^–10^. Then 100 μL of each diluted sample from 10^–4^ to 10^–10^ was plated onto the MRS agar medium and cultured in incubator at 37 ℃ for 48 h to get single colonies. The single colonies with different morphology were selected randomly from the petri dish and inoculated in the sterilized MRS liquid medium at 37 ℃ for about 12 h to get a different strain. The bacterial liquid was streaked to the MRS agar medium using the sterilized inoculation loop to get a single colony, the petri dishes were put upside down into the incubator at 37 ℃ for 48 h, thus completed one purification. Then, one well-isolated colony was inoculated in the sterilized MRS liquid medium at 37 ℃ for about 12 h to complete the isolation of colony purified bacteria. The optical density of the bacterial solution at 600 nm was measured to represent the proliferation of bacteria.

### Tolerance to acid, high temperature and antimicrobial susceptibility assay

The bacteria liquid with the same amount was inoculated in MRS at pH 2.0 for 4.5 h, or at pH 3.0, 3.5, and 4.0 for 8 h, respectively. The culture temperature was uniformly set at 37 ℃. Then the bacterial liquids were serially diluted to count on the MRS agar plates. The viable bacteria were expressed as the percentage of colony forming unit (CFU) at normal pH of 6.5.

The activated LAB liquid with the same amount of 10 mL was inoculated in MRS at 60, 70, and 80 ℃ for 160 s. while the control group had the same volume at 37 ℃. Then the bacterial liquids were serially diluted to count the number of viable bacteria. The viable bacteria were expressed as the percentage of CFU to control group.

A total of 20 different antibiotics frequently used in broiler chickens production were used in antimicrobial sensitivity trials by the disc plate method, and the susceptibility results were performed according to Clinical and Laboratory Standards Institute M100-S25 [48]. The bacteria were evenly inoculated in MRS agar plates, and the antibiotic disc was placed on the plate. The diameter of the inhibitory zones was measured after 48 h of culture at 37 ℃ in the incubator.

### Adherence, internalization and safety assays

The Caco-2 cells were inoculated on to cell culture plates and cultured in an incubator at 37 ℃ until they were full of single-layer cells. After that, the culture medium was replaced with a fresh medium containing live bacteria at 1 × 10^8^ CFU/mL, the supernatant of the medium was collected after 4.5 h of culture. The lactate dehydrogenase in the medium was measured using the lactate dehydrogenase kit (Nanjing Jiancheng Bioengineering Institute, Jiangsu, China). The lactate dehydrogenase activity was normalized according to the protein concentration of the supernatant.

The live bacteria were inoculated on the already confluent Caco-2 cells. Bacteria and cells were co-cultured for 3 h and the non-adhered bacteria were washed with PBS. The cells were then digested with trypsin and were serially diluted. Then the adhered bacteria were plated on MRS agar medium to count the number of viable bacteria. The adhesion rate was calculated based on the number of live bacteria as a percentage of the number of Caco-2 cells.

The Caco-2 cells were used for binding and internalization (gentamicin protection) assays as previously described. Briefly, 10^5^ cells per well were seeded in a 24-well plate. When the cells were confluent, the medium was changed to antibiotic-free medium and cultured for 12 h. The cells were co-cultured with *E. faecium* PNC01 at multiplicity of infection of 100 for 3 h, then the cells were washed with PBS three times to remove non-adherent *E. faecium* PNC01. *Salmonella* typhimurium was diluted in cell culture medium with a multiplicity of infection of 50 and co-cultured with the cells for 30 min. The cell wells were washed three times with PBS to remove non-internalized bacteria. Subsequently, the cells were cultured for 2 h in a fresh medium containing gentamicin at 100 μg/mL to kill extracellular bacteria, and then the concentration of gentamicin was changed to 10 μg/mL to restrict the growth of extracellular bacteria until the sample was collection. Infected cells were collected by PBS containing 0.1% (v/v) Triton-X100. Infected bacteria in the cells was counted by plating and counting using Xylose Lysine Deoxycholate Agar medium.

### Bacteriocin activity determination

The agar diffusion bioassay was modified to determine the selected bacterial bacteriocin activity.

After a single colony was expanded and cultured for 24 h, the supernatant was separated by centrifugation at 14,000 g for 5 min. The supernatant was filtered with a 0.22 um filter membrane and adjusted to pH 6.0 with sterile 1 M hydrochloric acid or 1 M sodium hydroxide to eliminate the inhibitory effect of organic acids on pathogenic bacteria. Subsequently, the supernatant was treated with 1 mg/mL catalase at 25 ℃ for 30 min to remove the inhibitory effect of hydrogen peroxide on bacteria. Five pathogenic bacteria were used to detect the bacteriocin activity of *E. faecium* PNC01, including *Staphylococcus* aureus ATCC-26003, *Escherichia* coli CICC10899, *Salmonella* enterica WX29, *Salmonella* typhimurium SL344 and *Salmonella* pullorum C79-13. The pathogenic bacteria were inoculated on LB agar culture plate at 10^5^ CFU/mL. 50ul of the supernatant and its twofold dilution were added to the Oxford cup placed on the culture plate. The antibacterial activity (AU) of bacteriocins is defined as 2^n^ × 1000 μL/50 μL, where n is the reciprocal of the highest dilution of supernatant that inhibited the pathogenic bacteria. Detailed steps bacteriocin activity was determined by the previous description [49].

### Identification of LAB

The DNA of the selected bacteria was extracted by DNA Extraction Kits (Tiangen Biochemical Technology Co., Ltd., Beijing, China) according to the instruction of manufacturer. The V1–V9 region of the 16S rRNA gene was amplified by PCR using universal primers (27F: 5'-AGAGTTTGATCCTGGCTCAG-3' and 1525R: 5'-AGAAAGGAGGTGATCCAGCCC-3'). The reaction conditions for PCR were 95 ℃ for 5 min, followed by 35 cycles at 90 ℃ for 30 s, 60 ℃ for 30 s and 72 ℃ for 90 s. The PCR products were sequenced by Sangon Biotech Co., Ltd. (Shanghai, China). The data were matched with the available data in GenBank.

### Draft genome sequence of bacterium

The genomic DNA isolated from *E. faecium* PNC01 for animal study was sequenced on the HiSeq4000 (Illumina, Inc., San Diego, CA) to generate paired-end 150 bp reads. The adapter sequences in the raw reads were trimmed using Trimmomatic v0.36 [50]. De novo assembly was performed using SPAdes [51]. The no-cording RNA was predicted using RNAmmer1.2 [52]. Gene functional annotation was carried out using prokka [53] and Gene Ontology with Pfam2go [54]. The metabolism prediction was analyzed by the antiSMASH 3.03 [55].

### Dietary processing and animal experimental design

The isolated *E. faecium* PNC01 was inoculated in the MRS medium at 37 ℃ for 48 h. The mix of arabic glue and skimmed milk powder with 1:1 ratio was added into the bacterial liquid at 200 g/L. Then the liquid was spray dried to get the bacterial powder. The final number of live bacteria in the powder was 5 × 10^9^ CFU/g.

The animal study was permitted by Chinese Agricultural University Laboratory Animal Welfare and Animal Experimental Ethical Committee (Approval number AW16109102-1). A total of 400 male one-day old Cobb broiler chickens were randomly assigned into 5 treatment groups and each group received one of the following 5 diets: control diet without addition (CON), control diet containing antibiotics of colistin sulfate at 40 mg/kg and zinc bacitracin at 20 mg/kg (Antibiotics), control diet containing low-dose of *E. faecium* PNC01 at 1 × 10^8^ CFU/kg feed (Low), basal diets containing median-dose of *E. faecium* PNC01 at 1 × 10^9^ CFU/kg feed (Medium), and basal diets containing high-dose of *E. faecium* PNC01 at 1 × 10^10^ CFU/kg feed (High). Every group had 8 replicate cages with 10 chickens per replicate pen. The composition of the control diet and nutrient levels are showed in Additional file 1: Table S3.

### Sample collection

The body weigh was measured at day 1, 7, 14, 21, 28, and 42, respectively, and the feed intake was recorded at day 7, 14, 21, 28, and 42. Eight broiler chickens were randomly selected for each treatment group that one chicken per repeating cage at day 21 and 42. The length of the duodenum, jejunum, ileum, and cecum was measured. The thymus, spleen and bursa of fabricius was weighed. The contents of each intestinal section were collected and stored at − 80 ℃ for future analysis. The mucous of each intestinal segment was scraped with glass slide. About 0.5 cm of each intestinal section was taken and fixed in 4% (w/v) paraformaldehyde solution for observation of intestinal morphology.

### Jejunal and ileal morphology

The fixed jejunal and ileal tissue was dehydrated by gradient ethanol and embedded in paraffin. The tissue was cut into 0.5 μm thickness. For each animal, ten villus height and crypt depth of each jejunum and ileum were measured by a light microscope. At the same time, their ratio of villus height to crypt depth was calculated.

### 16sDNA sequencing of intestinal bacteria and data analysis

On day 21, five chickens were randomly selected from the CON and the Medium groups, and the cecal contents were collected and stored at − 80 ℃ after rapid freezing by liquid nitrogen. The DNA from the cecal contents was isolated by DNA Extraction Kits (Tiangen Biochemical Technology Co., Ltd., Beijing, China). The V3–V4 region amplication of the 16S rRNA gene was performed using the following primers 338F: 5'-ACTCCTACGGGAGGCAGCAG-3' and 806R: 5'-GGACTACHVGGGTWTCTAAT-3'. Each sample was labeled with a specific barcode via PCR. All the samples were mixed with an equal amount of PCR product to create a library. The sequence was carried out by PE300 on an Illumina MiSeq platform. The sequences data were analyzed using the Quantitative Insights Into Microbial Ecology V1.9 (QIIME). The detailed process for diversity indices and PCoA analysis were referred to the previous method [11]. All the 16 s rRNA gene sequencing data are submitted to the Sequence Read Archive at NCBI under the BioProject number PRJNA627372.

### Statistical analysis

The data were analyzed using one-way ANOVA procedure of SAS 9.0 software. The significant differences among the five treatment groups were compared using Duncan’s multiple comparison test and the significant level was set at *P* < 0.05.

## Supplementary Information

**Additional file 1:** **Table S1.** Characteristic morphology of colony and cell of isolated lactic acid bacteria from chickens. **Table S2.** Effect of antibiotics and *Enterococcus faecium *PNC01 on immune organ index of broiler chickens. **Table S3.** Composition and nutrient levels of the experimental basal diet. **Figure S1.** The phylogenetic tree analysis shown *Enterococcus faecium *PNC01 was significantly different from other known *Enterococcus faecium*. **Figure S2.** The subsystem description of *Enterococcus faecium *PNC01*.* **Figure S3.** Effect of antibiotics and *Enterococcus faecium *PNC01 on jejunal and ileal morphology of broiler chickens. **Figure S4.** Effect of antibiotics and *Enterococcus faecium *PNC01 on the intestinal lactic acid bacteria of broiler chickens.

## Data Availability

All data generated or analyzed during this study are included in this published article.

## Abbreviations

LAB: Lactic acid bacteria
MRS: Man rogosa sharpe
FCR: Feed conversion rate
PBS: Phosphate buffered saline
PCoA: Principal coordinate analysis

## References

[1] Blaser MJ (2016). Antibiotic use and its consequences for the normal microbiome. Science.

[2] Laxminarayan R, Van Boeckel T, Frost I, Kariuki S, Khan EA, Limmathurotsakul D (2020). The Lancet Infectious Diseases Commission on antimicrobial resistance: 6 years later. Lancet Infect Dis.

[3] Awasthi MK, Chen H, Liu T, Awasthi SK, Wang Q, Ren X (2019). Respond of clay amendment in chicken manure composts to understand the antibiotic resistant bacterial diversity and its correlation with physicochemical parameters. J Clean Prod.

[4] Zhou S, Zhu D, Giles M, Daniell T, Neilson R, Yang X (2020). Does reduced usage of antibiotics in livestock production mitigate the spread of antibiotic resistance in soil, earthworm guts, and the phyllosphere?. Environ Int.

[5] Walsh TR, Wu Y (2016). China bans colistin as a feed additive for animals. Lancet Infect Dis.

[6] Hu Y, Cheng H, Tao S (2017). Environmental and human health challenges of industrial livestock and poultry farming in China and their mitigation. Environ Int.

[7] Wu Y, Zhen W, Geng Y, Wang Z, Guo Y (2019). Effects of dietary *Enterococcus faecium* NCIMB 11181 supplementation on growth performance and cellular and humoral immune responses in broiler chickens. Poultry Sci.

[8] Bodirsky BL, Popp A, Lotze-Campen H, Dietrich JP, Rolinski S, Weindl I (2014). Reactive nitrogen requirements to feed the world in 2050 and potential to mitigate nitrogen pollution. Nat Commun.

[9] Van Immerseel F, Eeckhaut V, Moore RJ, Choct M, Ducatelle R (2017). Beneficial microbial signals from alternative feed ingredients: a way to improve sustainability of broiler production?. Microb Biotechnol.

[10] Bäumler AJ, Sperandio V (2016). Interactions between the microbiota and pathogenic bacteria in the gut. Nature.

[11] He Y, Yang Y, Dong Y, Yan C, Zhang B (2019). The effects of flavomycin and colistin sulfate pre-treatment on ileal bacterial community composition, the response to *Salmonella* typhimurium and host gene expression in broiler chickens. Microorganisms.

[12] Lunedo R, Fernandez-Alarcon MF, Carvalho FMS, Furlan LR, Macari M (2014). Analysis of the intestinal bacterial microbiota in maize- or sorghum-fed broiler chickens using real-time PCR. Brit Poultry Sci.

[13] Mohammadi Ziarat M, Kermanshahi H, Nasiri Mogaddam H, Majidzadeh HR (2020). Performance of an *Escherichia* coli phytase expressed in *Lactococcus* lactis on nutrient retention, bone traits and intestinal morphology in broiler chickens. J Anim Physiol An N.

[14] Liu Q, Yu Z, Tian F, Zhao J, Zhang H, Zhai Q, Chen W (2020). Surface components and metabolites of probiotics for regulation of intestinal epithelial barrier. Microb Cell Fact.

[15] Onrust L, Ducatelle R, Van Driessche K, De Maesschalck C, Vermeulen K, Haesebrouck F (2015). Steering endogenous butyrate production in the intestinal tract of broilers as a tool to improve gut health. Front Vet Sci.

[16] Franz CM, Huch M, Abriouel H, Holzapfel W, Gálvez A (2011). *Enterococci* as probiotics and their implications in food safety. Int J Food Microbiol.

[17] Kreuzer-Redmer S, Bekurtz JC, Arends D, Bortfeldt R, Kutz-Lohroff B, Sharbati S (2016). Feeding of *Enterococcus faecium* NCIMB 10415 leads to intestinal miRNA-423-5p-Induced regulation of immune-relevant genes. Appl Environ Microb.

[18] Hwanhlem N, Ivanova T, Biscola V, Choiset Y, Haertlé T (2017). Bacteriocin producing *Enterococcus faecalis* isolated from chicken gastrointestinal tract originating from Phitsanulok, Thailand: Isolation, screening, safety evaluation and probiotic properties. Food Control.

[19] Šikić Pogačar M, Langerholc T, Mičetić-Turk D, Možina SS, Klančnik A (2020). Effect of *Lactobacillus* spp. on adhesion, invasion, and translocation of *Campylobacter jejuni* in chicken and pig small-intestinal epithelial cell lines. Bmc Vet Res.

[20] Xie S, Zhao S, Jiang L, Lu L, Yang Q, Yu Q (2019). *Lactobacillus**reuteri* stimulates intestinal epithelial proliferation and induces differentiation into goblet cells in young chickens. J Agr Food Chem.

[21] Adhikari B, Kwon YM (2017). Characterization of the culturable subpopulations of *Lactobacillus* in the chicken intestinal tract as a resource for probiotic development. Front Microbiol.

[22] Zheng S, Di G, Liu S, Wang Q, Liu S, Wang R (2019). A newly isolated human intestinal bacterium strain capable of deglycosylating flavone C-glycosides and its functional properties. Microb Cell Fact.

[23] Timmerman HM, Veldman A, van den Elsen E, Rombouts FM, Beynen AC (2006). Mortality and growth performance of broilers given drinking water supplemented with chicken-specific probiotics. Poultry Sci.

[24] Brandt KK, Amezquita A, Backhaus T, Boxall A, Coors A, Heberer T (2015). Ecotoxicological assessment of antibiotics: a call for improved consideration of microorganisms. Environ Int.

[25] Patnaik R, Louie S, Gavrilovic V, Perry K, Stemmer W, Ryan CM, Del Cardayre S (2002). Genome shuffling of *Lactobacillus* for improved acid tolerance. Nat Biotechnol.

[26] Kailasapathy K (2002). Microencapsulation of probiotic bacteria: technology and potential applications. Curr Issues Intest Microbiol.

[27] Kobierecka PA, Wyszyńska AK, Aleksandrzak Piekarczyk T, Kuczkowski M, Tuzimek A, Piotrowska W (2017). In vitro characteristics of Lactobacillus spp. strains isolated from the chicken digestive tract and their role in the inhibition of Campylobacter colonization. Microbiologyopen.

[28] Jiang S, Cai L, Lv L, Li L (2021). *Pediococcus pentosaceus*, a future additive or probiotic candidate. Microb Cell Fact.

[29] Zhou X, Wang J, Lu C, Liao Q, Gudda FO, Ling W. Antibiotics in animal manure and manure-based fertilizers: occurrence and ecological risk assessment. Chemosphere 2020:127006. 10.1016/j.chemosphere.2020.127006 32417517

[30] Cui Y, Wang S, Ding S, Shen J, Zhu K (2020). Toxins and mobile antimicrobial resistance genes in *Bacillus* probiotics constitute a potential risk for one health. J Hazard Mater.

[31] Kaktcham PM, Zambou NF, Tchouanguep FM, El-Soda M, Choudhary MI (2012). Antimicrobial and safety properties of *Lactobacilli* isolated from two cameroonian traditional fermented foods. Sci Pharm.

[32] Mack DR, Ahrne S, Hyde L, Wei S, Hollingsworth MA (2003). Extracellular MUC3 mucin secretion follows adherence of Lactobacillus strains to intestinal epithelial cells in vitro. Gut.

[33] Popović N, Djokić J, Brdarić E, Dinić M, Terzić-Vidojević A, Golić N, Veljović K (2019). The influence of heat-killed *Enterococcus faecium* BGPAS1–3 on the tight junction protein expression and immune function in differentiated Caco-2 Cells infected with Listeria monocytogenes ATCC 19111. Front Microbiol.

[34] Liu L, Mei Q, Liu L, Zhang F, Liu Z, Wang Z, Wang R (2005). Protective effects of *Rheum* tanguticum polysaccharide against hydrogen peroxide-induced intestinal epithelial cell injury. World J Gastroentero.

[35] Liu MJ, van Enckevort F, Siezen RJ (2005). Genome update: lactic acid bacteria genome sequencing is booming. Microbiol-Sgm.

[36] Collins FWJ, O’Connor PM, O’Sullivan O, Gomez-Sala B, Rea MC, Hill C, Ross RP (2017). Bacteriocin Gene-Trait matching across the complete Lactobacillus Pan-genome. Sci Rep Uk.

[37] Qian Y, Song K, Hu T, Ying T (2018). Environmental status of livestock and poultry sectors in China under current transformation stage. Sci Total Environ.

[38] Jin LZ, Ho YW, Abdullah N, Ali MA, Jalaludin S (1998). Effects of adherent *Lactobacillus* cultures on growth, weight of organs and intestinal microflora and volatile fatty acids in broilers. Anim Feed Sci Tech.

[39] Ma Y, Wang W, Zhang H, Wang J, Zhang W, Gao J (2018). Supplemental Bacillus subtilis DSM 32315 manipulates intestinal structure and microbial composition in broiler chickens. Sci Rep-Uk.

[40] Röhe I, Boroojeni FG, Zentek J (2017). Effect of feeding soybean meal and differently processed peas on intestinal morphology and functional glucose transport in the small intestine of broilers. Poultry Sci.

[41] Darabighane B, Zarei A, Shahneh AZ, Mahdavi A (2011). Effects of different levels of Aloe vera gel as an alternative to antibiotic on performance and ileum morphology in broilers. Ital J Anim Sci.

[42] Wu Y, Zhen W, Geng Y, Wang Z, Guo Y (2019). Pretreatment with probiotic *Enterococcus faecium* NCIMB 11181 ameliorates necrotic enteritis-induced intestinal barrier injury in broiler chickens. Sci Rep-Uk.

[43] Wei H, Li X, Tang L, Yao H, Ren Z, Wang C, et al. 16S rRNA gene sequencing reveals the relationship between gut microbiota and ovarian development in the swimming crab *Portunus trituberculatus*. Chemosphere 2020:126891. 10.1016/j.chemosphere.2020.126891 32957291

[44] Fang S, Chen X, Zhou L, Wang C, Chen Q, Lin R (2019). Faecal microbiota and functional capacity associated with weaning weight in meat rabbits. Microb Biotechnol.

[45] Rodrigues DR, Briggs W, Duff A, Chasser K, Murugesan R, Pender C (2020). Comparative effectiveness of probiotic-based formulations on cecal microbiota modulation in broilers. PLoS ONE.

[46] Rodrigues DR, Briggs W, Duff A, Chasser K, Murugesan R, Pender C (2020). Cecal microbiome composition and metabolic function in probiotic treated broilers. PLoS ONE.

[47] Lam K, Cheung PC (2019). Carbohydrate-based prebiotics in targeted modulation of gut microbiome. J Agr Food Chem.

[48] CLSI**.** Performance standards for antimicrobial susceptibility testing. In: Clinical and Laboratory Standards Institute Wayne, PA; 2017.

[49] Yang E, Fan L, Yan J, Jiang Y, Doucette C, Fillmore S, Walker B (2018). Influence of culture media, pH and temperature on growth and bacteriocin production of bacteriocinogenic lactic acid bacteria. AMB Express.

[50] Bolger AM, Lohse M, Usadel B (2014). Trimmomatic: a flexible trimmer for Illumina sequence data. Bioinformatics.

[51] Bankevich A, Nurk S, Antipov D, Gurevich AA, Dvorkin M, Kulikov AS (2012). SPAdes: a new genome assembly algorithm and its applications to single-cell sequencing. J Comput Biol.

[52] Lagesen K, Hallin P, Rødland EA, Stærfeldt H, Rognes T, Ussery DW (2007). RNAmmer: consistent and rapid annotation of ribosomal RNA genes. Nucleic Acids Res.

[53] Seemann T (2014). Prokka: rapid prokaryotic genome annotation. Bioinformatics.

[54] Bateman A, Coin L, Durbin R, Finn RD, Hollich V, Griffiths-Jones S (2004). The Pfam protein families database. Nucleic Acids Res.

[55] Weber T, Blin K, Duddela S, Krug D, Kim HU, Bruccoleri R (2015). antiSMASH 3.0—a comprehensive resource for the genome mining of biosynthetic gene clusters. Nucleic Acids Res.

