# Supplementary material for: Enterococcus faecium PNC01 isolated from the intestinal mucosa of chicken as an alternative for antibiotics to reduce feed conversion rate in broiler chickens
Source: Microb Cell Fact. 2021 Jun 28;20:122. doi: 10.1186/s12934-021-01609-z (PMC8240220; doi:10.1186/s12934-021-01609-z)
Supplement: Supplementary file 1 — Additional file 1: Table S1. Characteristic morphology of colony and cell of isolated lactic acid bacteria from chickens. Table S2. Effect of antibiotics and Enterococcus faecium PNC01 on immune organ index of broiler chickens. Table S3. Composition and nutrient levels of the experimental basal diet. Figure S1. The phylogenetic tree analysis shown Enterococcus faecium PNC01 was significantly different from other known Enterococcus faecium. Figure S2. The subsystem description of Enterococcus faecium PNC01. Figure S3. Effect of antibiotics and Enterococcus faecium PNC01 on jejunal and ileal morphology of broiler chickens. Figure S4. Effect of antibiotics and Enterococcus faecium PNC01 on the intestinal lactic acid bacteria of broiler chickens. [file 12934_2021_1609_MOESM1_ESM.docx]

Table S1. Characteristic morphology of colony and cell of isolated lactic acid bacteria from chickens

| Isolate Code | Proliferation efficacy^1^ | Colony morphology^2^ (Size) | Cell morphology^3^ |
| --- | --- | --- | --- |
| *Pediococcus pentosaceus1* | 1.031 | small | occur in pairs, short, small |
| *Pediococcus pentosaceus2* | 0.98 | small | occur in pairs, short, small |
| *Pediococcus pentosaceus3* | 0.864 | small | occur in chains, long, large |
| *Pediococcus pentosaceus4* | 0.758 | medium | occur in pairs, short, small |
| *Pediococcus pentosaceus5* | 0.685 | small | occur in pairs, short, small |
| *Pediococcus pentosaceus6* | 0.675 | medium | occur in pairs, short, small |
| *Pediococcus pentosaceus7* | 0.617 | small | occur in chains, long, large |
| *Pediococcus pentosaceus8* | 0.547 | small | occur in pairs, short, small |
| *Pediococcus pentosaceus9* | 0.531 | medium | occur in pairs, short, small |
| *Pediococcus pentosaceus10* | 0.517 | small | occur in pairs, short, small |
| *Pediococcus pentosaceus11* | 0.506 | big | occur in chains, long, large |
| *Pediococcus pentosaceus12* | 0.413 | small | occur in chains, long, large |
| *Pediococcus pentosaceus13* | 0.364 | small | occur in pairs, short, small |
| *Lactobacillus salivarius1* | 1.006 | small | Bacilli, short, small |
| *Lactobacillus salivarius2* | 0.969 | medium | Bacilli, short, small |
| *Lactobacillus salivarius3* | 0.889 | small | Bacilli, long, large |
| *Lactobacillus salivarius4* | 0.869 | medium | Bacilli, short, small |
| *Lactobacillus salivarius5* | 0.814 | big | Bacilli, short, small |
| *Lactobacillus salivarius6* | 0.812 | small | Bacilli, short, small |
| *Lactobacillus salivarius7* | 0.762 | small | Bacilli, short, small |
| *Lactobacillus salivarius8* | 0.733 | medium | Bacilli, short, small |
| *Lactobacillus salivarius9* | 0.698 | small | Bacilli, short, small |
| *Lactobacillus salivarius10* | 0.673 | small | Bacilli, short, small |
| *Lactobacillus salivarius11* | 0.669 | big | Bacilli, short, small |
| *Lactobacillus salivarius12* | 0.638 | small | Bacilli, short, small |
| *Lactobacillus salivarius13* | 0.621 | small | Bacilli, short, small |
| *Lactobacillus salivarius14* | 0.613 | small | Bacilli, short, small |
| *Lactobacillus salivarius15* | 0.595 | small | Bacilli, short, small |
| *Lactobacillus salivarius16* | 0.594 | big | Bacilli, short, small |
| *Lactobacillus salivarius17* | 0.581 | small | Bacilli, long, large |
| *Lactobacillus salivarius18* | 0.569 | small | Bacilli, short, small |
| *Lactobacillus salivarius19* | 0.565 | small | Bacilli, short, small |
| *Lactobacillus salivarius20* | 0.563 | big | Bacilli, short, small |
| *Lactobacillus salivarius21* | 0.562 | big | Bacilli, short, small |
| *Lactobacillus salivarius22* | 0.561 | small | Bacilli, long, large |
| *Lactobacillus salivarius23* | 0.560 | small | Bacilli, short, small |
| *Lactobacillus salivarius24* | 0.556 | small | Bacilli, short, small |
| *Lactobacillus salivarius25* | 0.552 | big | Bacilli, short, small |
| *Lactobacillus salivarius26* | 0.55 | small | Bacilli, long, large |
| *Lactobacillus salivarius27* | 0.536 | big | Bacilli, long, large |
| *Lactobacillus salivarius28* | 0.527 | small | Bacilli, short, small |
| *Lactobacillus salivarius29* | 0.526 | small | Bacilli, short, small |
| *Lactobacillus salivarius30* | 0.522 | small | Bacilli, short, small |
| *Lactobacillus salivarius31* | 0.495 | small | Bacilli, long, large |
| *Lactobacillus salivarius32* | 0.493 | small | Bacilli, long, large |
| *Lactobacillus salivarius33* | 0.49 | big | Bacilli, short, small |
| *Lactobacillus salivarius34* | 0.488 | small | Bacilli, short, small |
| *Lactobacillus salivarius35* | 0.469 | small | Bacilli, long, large |
| *Lactobacillus salivarius36* | 0.446 | small | Bacilli, long, large |
| *Lactobacillus salivarius37* | 0.423 | big | Bacilli, short, small |
| *Lactobacillus salivarius38* | 0.360 | small | Bacilli, long, large |
| *Lactobacillus salivarius39* | 0.349 | small | Bacilli, short, small |
| *Lactobacillus salivarius40* | 0.382 | medium | Bacilli, short, small |
| *Lactobacillus salivarius41* | 0.364 | medium | Bacilli, short, small |
| *Lactobacillus salivarius42* | 0.355 | small | Bacilli, long, large |
| *Lactobacillus salivarius43* | 0.343 | big | Bacilli, short, small |
| *Lactobacillus salivarius44* | 0.338 | small | Bacilli, short, small |
| *Lactobacillus salivarius45* | 0.328 | big | Bacilli, short, small |
| *Lactobacillus salivarius46* | 0.323 | medium | Bacilli, short, small |
| *Lactobacillus salivarius47* | 0.306 | small | Bacilli, short, small |
| *Lactobacillus salivarius48* | 0.297 | small | Bacilli, short, small |
| *Lactobacillus salivarius49* | 0.291 | small | Bacilli, long, large |
| *Lactobacillus salivarius50* | 0.281 | medium | Bacilli, short, small |
| *Lactobacillus salivarius51* | 0.280 | big | Bacilli, long, large |
| *Lactobacillus salivarius52* | 0.278 | small | Bacilli, long, large |
| *Lactobacillus salivarius53* | 0.276 | small | Bacilli, short, small |
| *Lactobacillus salivarius54* | 0.275 | small | Bacilli, short, small |
| *Lactobacillus salivarius55* | 0.264 | small | Bacilli, short, small |
| *Lactobacillus reuteri* | 0.999 | small | Bacilli, long, large |
| *Enterococcus faecium* | 1.001 | medium | Bacilli, short, small |

1. The proliferation efficiency is the OD600 of the bacterial solution when a single colony is inoculated for 12 h.

2. The pigmentation of all isolated bacteria was milky white, the shape was circular, and the edge was entire.

3. The Gram stain of all isolated bacteria was positive.

Table S2. Effect of antibiotics and *Enterococcus faecium* PNC01 on immune organ index of broiler chickens.

| Treatment^1^ | Thymus (g/kg body weight) | | Spleen (g/kg body weight) | | Bursa of fabricius (g/kg body weight) | |
| --- | --- | --- | --- | --- | --- | --- |
|  | 21d | 42d | 21d | 42d | 21d | 42d |
| CON | 1.22 | 2.3 | 1.3 | 0.82 | 0.6 | 0.37 |
| Antibiotics | 1.16 | 2.14 | 1.08 | 0.94 | 0.65 | 0.35 |
| Low | 1.51 | 2.1 | 1.36 | 0.87 | 0.63 | 0.33 |
| Medium | 1.29 | 2.56 | 1.28 | 0.85 | 0.66 | 0.36 |
| High | 1.46 | 2.46 | 1.28 | 1 | 0.72 | 0.35 |
| SEM | 0.061 | 0.08 | 0.049 | 0.334 | 0.027 | 0.019 |
| *P* value | 0.303 | 0.292 | 0.497 | 0.459 | 0.732 | 0.985 |

1 Treatment: CON, control diet without addition (CON); Antibiotics, control diet containing antibiotics of colistin sulfate at 40mg/kg and zinc bacitracin at 20mg/kg; Low, control diet containing *Enterococcus faecium* at 1 × 10^8^ CFU/kg feed; Medium, basal diets containing *Enterococcus faecium* at 1 × 10^9^ CFU/kg feed; High, and basal diets containing *Enterococcus faecium* at 1 × 10^10^ CFU/kg feed.

Table S3. Composition and nutrient levels of the experimental basal diet.

| Ingredients (%) | 1-14 day | 15-28 day | 29-42 day |
| --- | --- | --- | --- |
| Corn | 55.4 | 55.35 | 61.55 |
| Soybean meal | 37.72 | 37.67 | 31.04 |
| Soy oil | 2.6 | 2.6 | 3.3 |
| Calcium hydrogen phosphate | 1.96 | 1.96 | 1.77 |
| Limestone | 1.18 | 1.18 | 1.07 |
| DL-Methionine | 0.22 | 0.22 | 0.12 |
| L-Lys HCL | 0.15 | 0.15 | 0.21 |
| Vitamin premix^1^ | 0.02 | 0.02 | 0.02 |
| Mineral premix^2^ | 0.2 | 0.2 | 0.2 |
| Sodium chloride | 0.3 | 0.3 | 0.3 |
| Choline chloride(50%) | 0.13 | 0.13 | 0.2 |
| Antioxidant | 0.02 | 0.02 | 0.02 |
| Maifanite | 0.1 | 0.1 | 0.2 |
| Nutrient levels |  |  |  |
| Metabolic energy, calculated (kcal/kg) | 2906.22 | 2903.45 | 3011.69 |
| Crude protein(%) | 21.33 | 21.31 | 19 |
| Lysine(%) | 1.18 | 1.17 | 1.07 |
| Methionine (%) | 0.56 | 0.56 | 0.43 |
| Calcium (%) | 1 | 1 | 0.9 |
| Available phosphorus (%) | 0.45 | 0.45 | 0.42 |
| Total phosphorus (%) | 0.7 | 0.7 | 0.65 |

1 The vitamine premix provides the following per kg in the diet: vitamin A: 9500 IU; vitamin D3: 62.5 µg; vitamin E: 30 IU; vitamin K3: 2.65 mg; vitamin B1: 2 mg; vitamin B6 6 mg; vitamin B12: 0.025 mg; biotin: 0.0325 mg; folic acid: 1.25 mg; pantothenic acid: 12 mg; nicotinic acid: 50 mg.

2 Mineral premix provided per kg of complete diet: copper: 8 mg; ferrum: 80 mg; manganese: 100 mg; selenium: 0.15 mg; iodine: 0.35 mg.


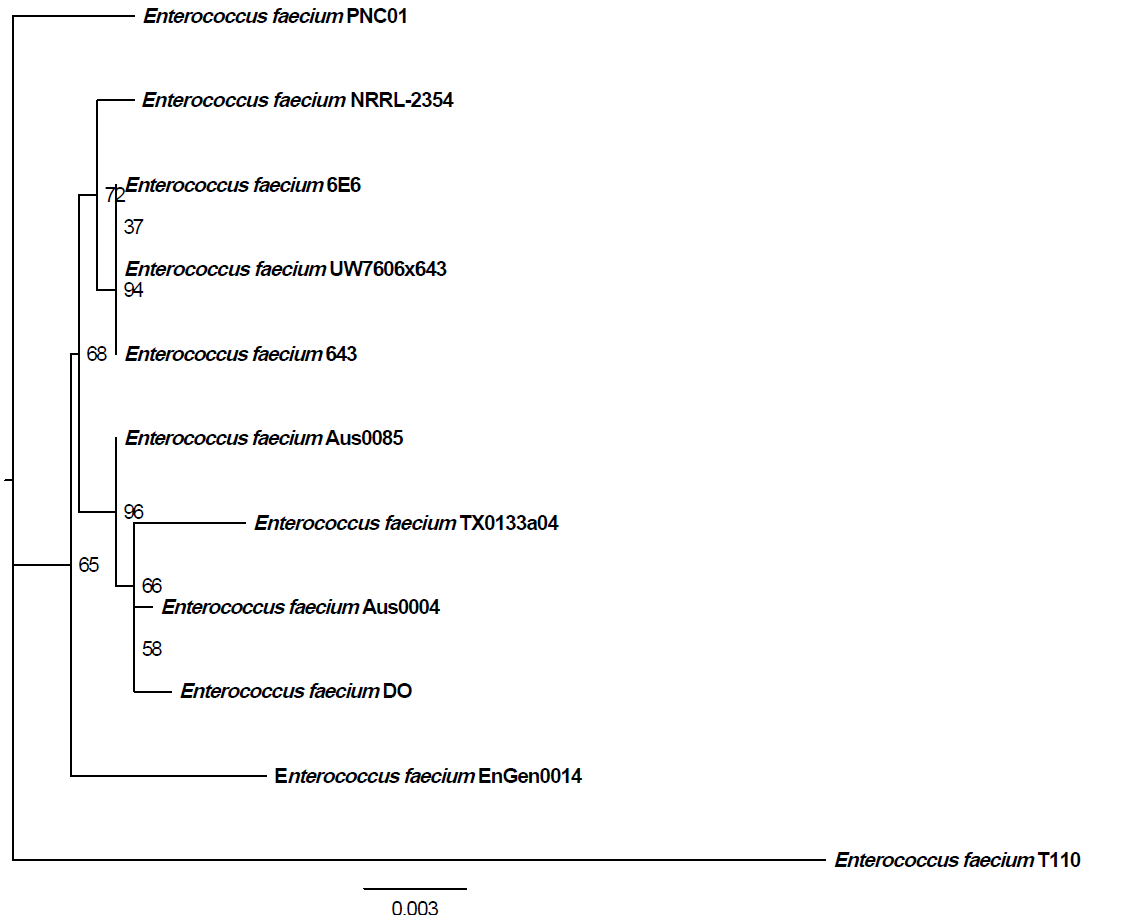


Figure S1. The phylogenetic tree analysis shown *Enterococcus faecium* PNC01 was significantly different from other known *Enterococcus faecium.*


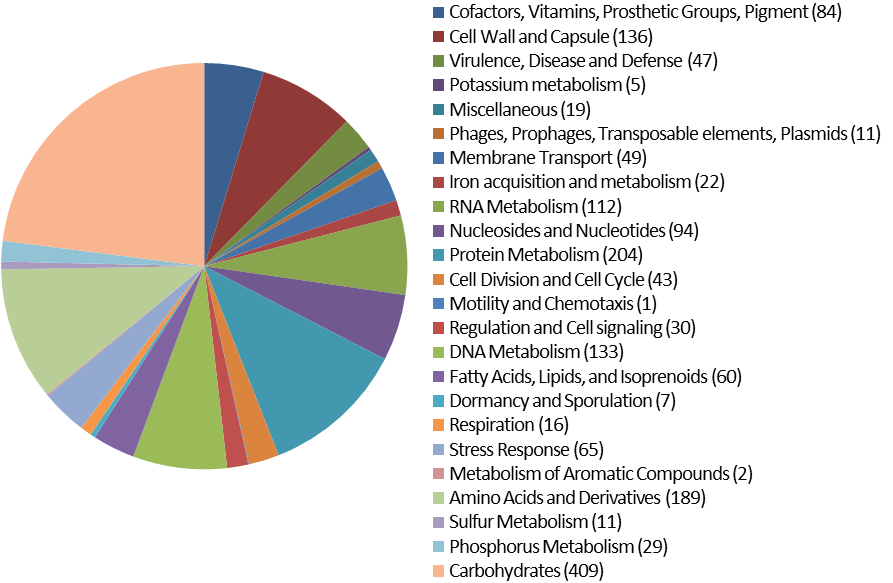


Figure S2. The subsystem description of *Enterococcus faecium* PNC01*.*

*
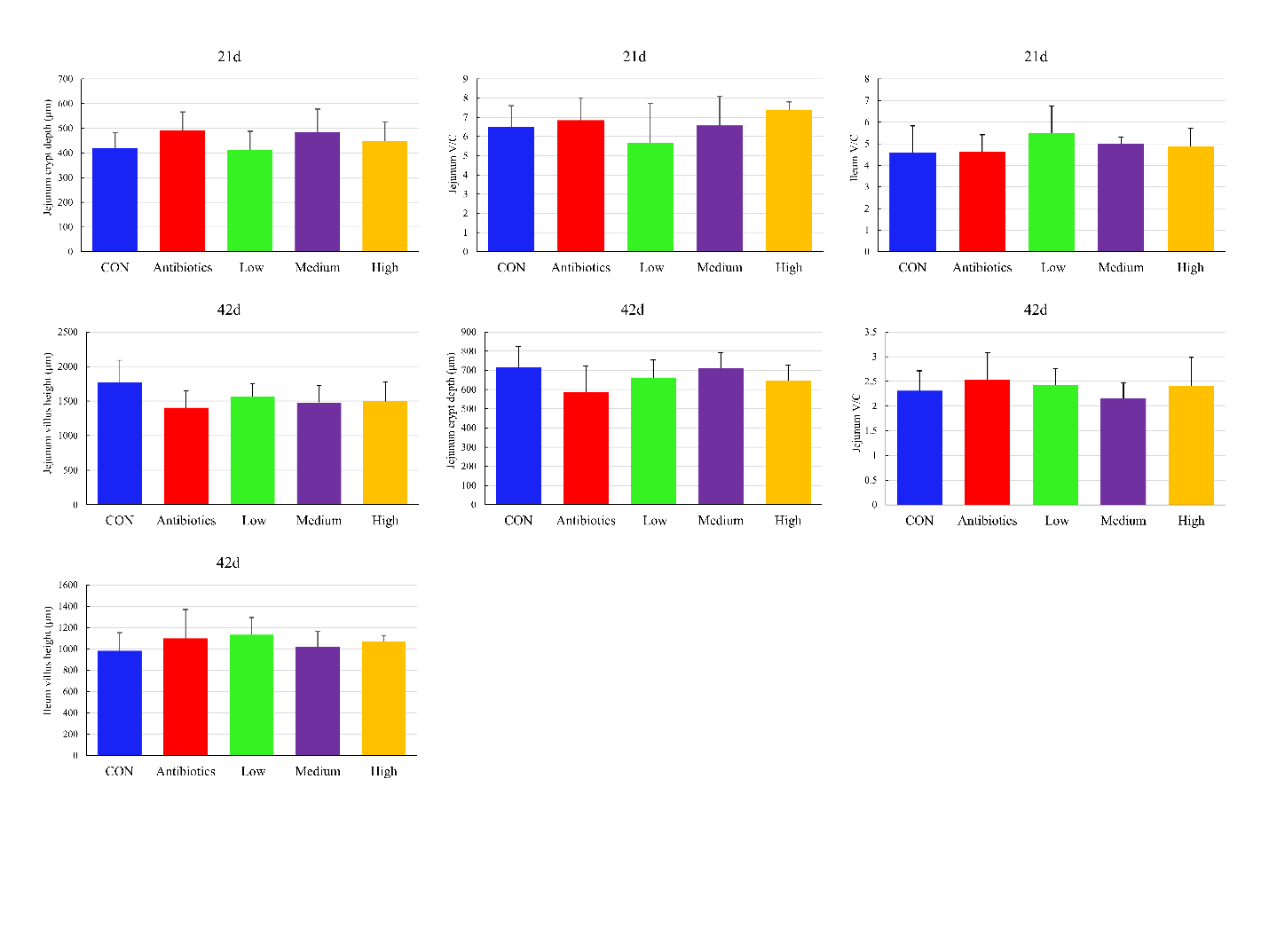
*

Figure S3 Effect of antibiotics and *Enterococcus faecium* PNC01 on jejunal and ileal morphology of broiler chickens.


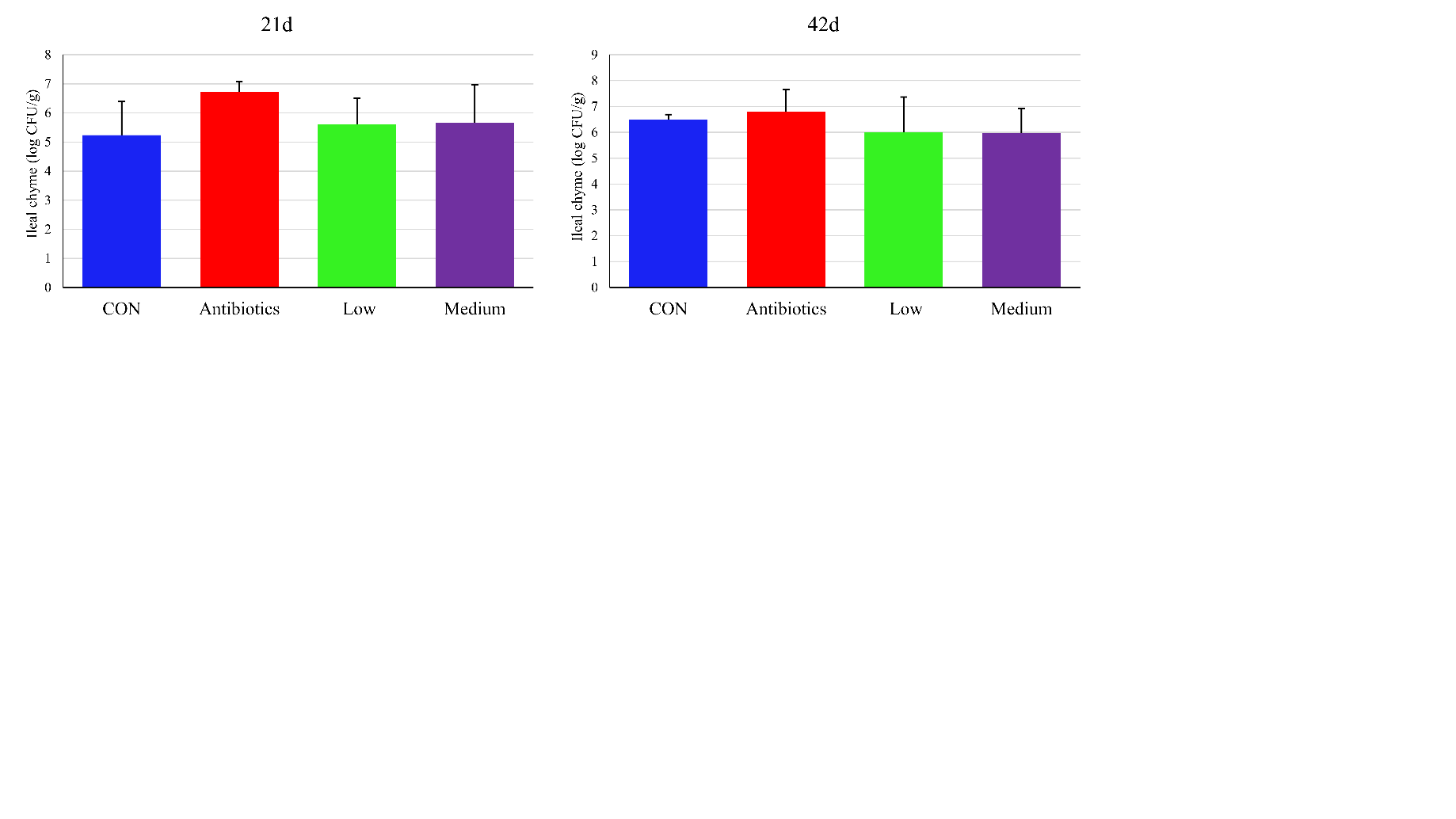


Figure S4 Effect of antibiotics and *Enterococcus faecium* PNC01 on the intestinal lactic acid bacteria of broiler chickens.
